# Significance of the lobe-specific emphysema index to predict prolonged air leak after anatomical segmentectomy

**DOI:** 10.1371/journal.pone.0224519

**Published:** 2019-11-05

**Authors:** Duk Hwan Moon, Chul Hwan Park, Du-Young Kang, Hye Sun Lee, Sungsoo Lee

**Affiliations:** 1 Department of Thoracic and Cardiovascular Surgery, Gangnam Severance Hospital, Yonsei University College of Medicine, Seoul, Republic of Korea; 2 Department of Radiology and Research Institute of Radiological Science, Gangnam Severance Hospital, Yonsei University College of Medicine, Seoul, Republic of Korea; 3 Department of Cardiovascular and Thoracic Surgery, Kangbuk Samsung Hospital, Sungkyunkwan University School of Medicine, Seoul, Republic of Korea; 4 Biostatics Collaboration Unit, Yonsei University College of Medicine, Seoul, Republic of Korea; Baylor College of Medicine, UNITED STATES

## Abstract

Prolonged air leak (PAL) is a major complication of pulmonary resection. Emphysema quantification with computed tomography is regarded as an important predictor of PAL for patients undergoing lobectomy. Therefore, we investigated whether this predictor might be applicable for segmentectomy. Herein, we characterized the factors that influence PAL in early stage lung cancer patients undergoing anatomical segmentectomy. Forty-one patients who underwent anatomical segmentectomy for early lung cancer between January 2014 and July 2017 were included for analysis. Several baseline and surgical variables were evaluated. In particular, the emphysema index (EI, %) and lobe-specific emphysema index (LEI, %) were assessed by using three-dimensional volumetric CT scan. PAL was observed in 13 patients (31.7%). There were statistically significant differences in DLCO (97.3% ± 18.3% vs. 111.7% ± 15.9%, p = 0.014), EI (4.61% ± 4.66% vs. 1.17% ± 1.76%, p = 0.023), and LEI (5.81% ± 5.78% vs. 0.76% ± 1.17%, p = 0.009) between patients with and without PAL. According to logistic regression analysis, both EI and LEI were significantly associated with PAL (p = 0.028 and p < 0.001, respectively). We found that EI and LEI significantly influenced the development of PAL after pulmonary resection. In particular, LEI showed stronger association with PAL, compared with EI, suggesting the importance of LEI in the prediction of PAL after anatomical segmentectomy.

## Introduction

Prolonged air leak (PAL) is one of the most common complications of lung resection [1–4]. It is associated with prolonged hospital stay, as well as increased hospital costs due to the increased likelihood of developing serial complications, including empyema, deep vein thrombosis, and respiratory infections [1,3,5]. Currently, factors known to be predictive of PAL resemble the features of the emphysema phenotype of chronic obstructive pulmonary disease (COPD); these factors include male gender, low body mass index (BMI), low diffusing capacity for carbon monoxide (DLCO), and Medical Research Council (MRC) dyspnea scale < 1 [2,3,6]. Other factors, including surgical technique, surgical site, and pleural adhesion, have also been identified [3].

Among the various predictors, emphysema quantification with computed tomography (CT) has been regarded as an important predictor of PAL for patients undergoing lobectomy [7]. Hence, we investigated whether this predictor might be applicable for segmentectomy. Recently, with the increased use of chest CT in many institutions, there has been an increasing need to perform sublobar resection—a type of anatomical segmentectomy—because of the increase in the detection of early-stage lung cancer and ground-glass opacity nodules (GGN) [8]. We suspect that in this case, the lobe-specific emphysema index (LEI), rather than the whole-lung emphysema index (EI), may be more useful. Hence, the purposes of this study were twofold: (1) to analyze factors predictive for the development of PAL in patients with early stage lung cancer who were undergoing anatomical segmentectomy, and (2) to evaluate which of the two indexes—LEI or EI—exhibits greater predictive significance.

## Materials and methods

This study was approved by our Institutional Ethics Committee/Review Board (Gangnam Severance Hospital); the requirement for informed consent was waived due to the retrospective nature of the analyses.

**Fig 1:**
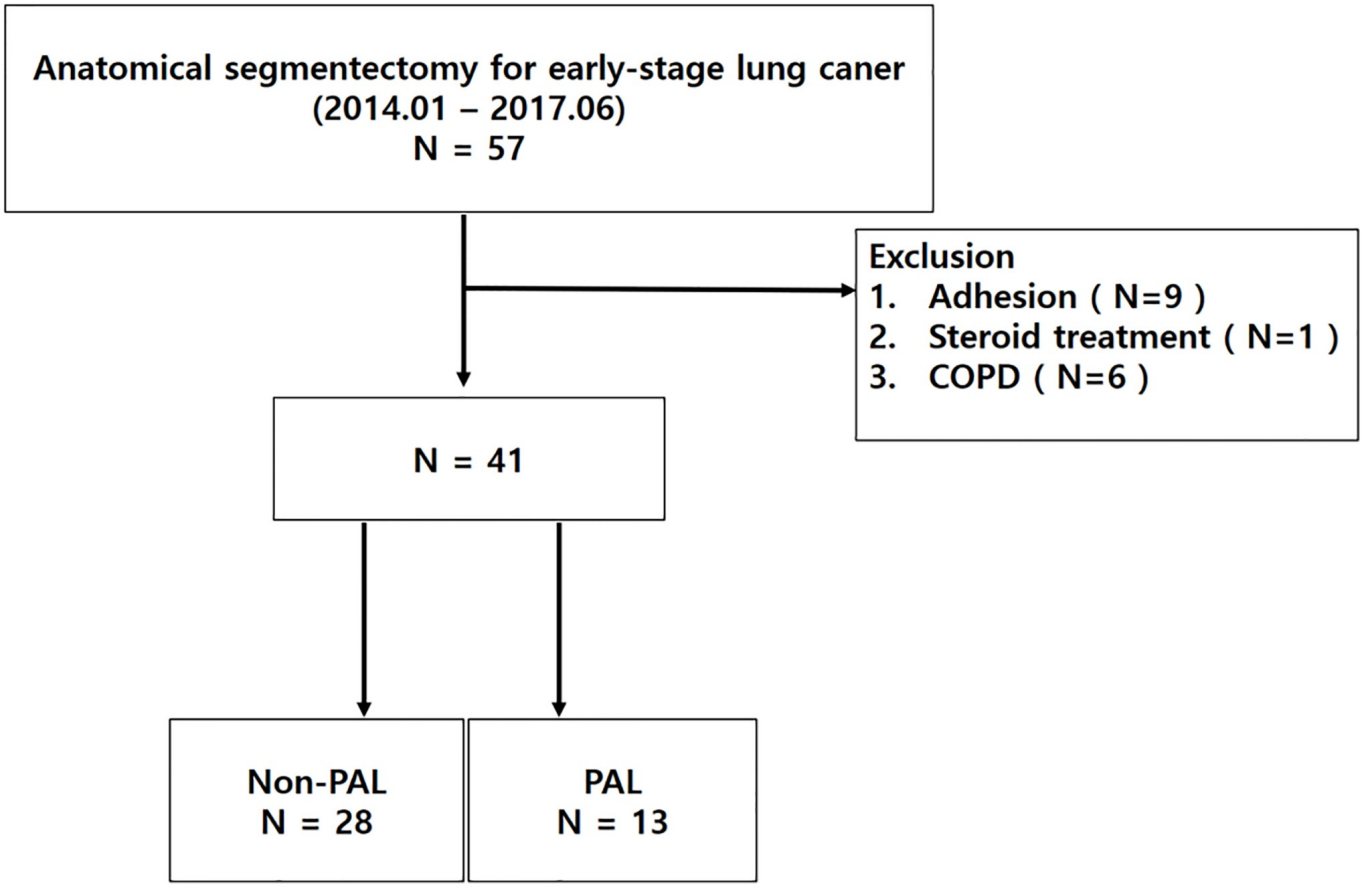
Flowchart for patient selection.

This retrospective study recruited early stage lung cancer patients who underwent anatomical segmentectomy between January 2014 and July 2017 at a single institution. We excluded patients who received radiation treatment, steroid treatment, and/or had pleural adhesion (Fig 1). We defined PAL as the presence of persistent air leak for more than 5 consecutive days or the requirement for chemical pleurodesis. Medical records, including patient characteristics and operative details, were reviewed and analyzed.

### Surgical technique

All anatomical segmentectomies were performed via video-assisted thoracoscopic surgery (VATS) under single-lung ventilation with a dual-lumen endotracheal tube. Fiberoptic bronchoscopy was routinely performed to ensure that the segmental bronchus was free of disease. Three incisions were made to perform VATS segmentectomy. The primary incision (3.5 cm) was made at the anterior-to mid-axillary line of the 5^th^ intercostal space (ICS); the second incision was made at the anterior axillary line of the 7^th^ ICS for 10-mm scope insertion; and the third incision was made at the mid-axillary line of the 7^th^ ICS as the 12-mm instrument port. A thoracoscopy was inserted to inspect the thoracic cavity and assess the viability of the segmentectomy procedure. When viability was confirmed, hilar dissection was performed to expose the segmental pulmonary vein, which was then dissected and divided by using an endoscopic stapler. The segmental artery and segmental bronchus–in that order–were dissected and divided with an endoscopic stapler. All visible lymph nodes in the operative field were resected. Then, the parenchyma was also divided with an endoscopic stapler after temporarily reinflating the lung to define the segmental anatomy. Segmentectomy was not performed in patients with tumors less than 1 cm in size in the intersegmental plane. To avoid wound seeding, the resected specimen was removed in a sterile, protective, plastic bag before performing mediastinal lymph node dissection. No surgical materials were used to cover the damaged tissues, such as polyglycolic acid (PGA) sheets or fibrin glue.

### CT protocol and image analysis

**Fig 2:**
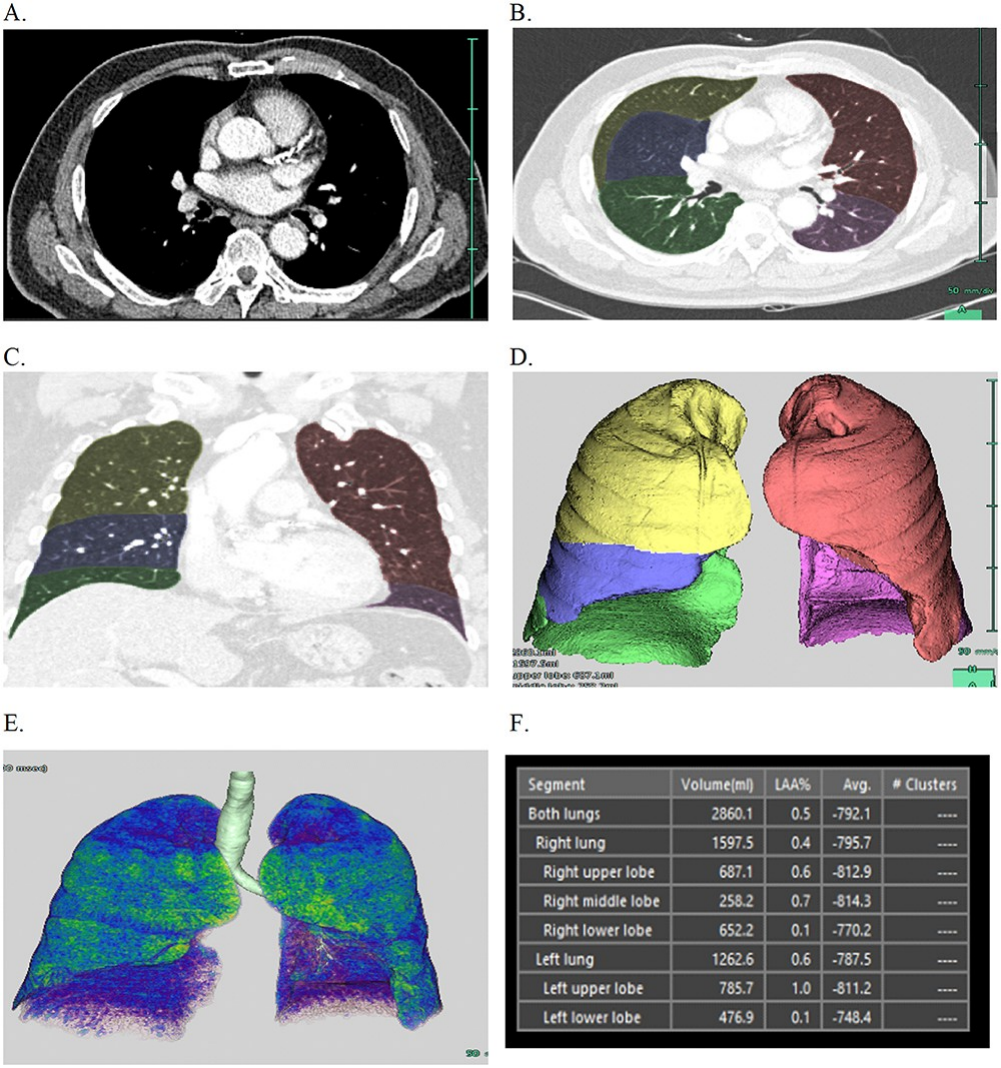
Process of obtaining emphysema index and lobe-specific emphysema index. Computed tomography image (A) is converted to a three-dimensional volume rendering image using a commercially available software, Synapse 3D Vincent^™^. Lobes are divided via the automatic segmentation technique and manual correction (B, C), and the volume of each lobe is produced (D). Lobe-specific emphysema index for each lobe is obtained by defining the emphysema index with an attenuation value lower than -950 Hounsfield unit (E, F).

Chest CT scans were performed with one of the following three scanners: Somatom Sensation 16, Somatom Sensation 64, or Somatom Definition AS+ (all Siemens Medical Solutions, Erlangen, Germany). Participants were scanned in the supine position from the lung apex to the adrenal glands during breath-holding at the end of inspiration. Volumetric CT scans were obtained from all patients with the following parameters: 120 kVp, 100–150 mAs, pitch of 1, 1.0-mm section thickness, and contiguous section interval. From CT data, lungs and lobes were semi-automatically segmented with a commercially available pulmonary dedicated software package (Synapse 3D Vincent, Fuji Film Co., Ltd., Tokyo, Japan). The EI was defined as the percentage of all lung voxels with attenuation values lower than -950 HU [9], and was measured from each lung (right lung and left lung); LEI was also measured in each lung (right upper lobe, right middle lobe, right lower lobe, left upper lobe, and left lobe) (Fig 2).

### Statistical analysis

Categorical variables were presented as a number (%), and continuous variables were presented as mean ± standard deviation (SD). Categorical variables between the PAL-group and non-PAL group were compared by using the chi-squared test (or Fisher’s exact test); continuous variables were compared by using Student’s t-test. For both univariate and multivariate analyses, a logistic regression analysis model was used to determine risk factors of PAL; odds ratios (ORs) and 95% confidence intervals (95% CIs) were calculated with the non-PAL group as the reference. Variables with a p-value ≤ 0.5 in univariate analyses were candidates for the multivariate models. Two multivariate models were considered to avoid multicollinearity of the EI and LEI variables. The first model included EI, while the second model included LEI. To compare the performances of EI and LEI, receiver operating characteristic (ROC) curves were constructed and the area under the curve (AUC) was compared between the two curves. Comparisons were made by using the Delong methods for statistical significance of AUC. Additionally, the optimal cut-off value for predicting PAL was determined based on the value that maximized the Youden index (sensitivity + specificity-1). All statistical analyses were performed by using SAS (version 9.3, SAS Inc., Cary, NC, USA). A two-sided *p* value of less than 0.05 was considered statistically significant.

## Results

All included patients (n = 41) were divided into two groups: the PAL group (n = 13) and non-PAL group (n = 28). The mean age was 63.9 ± 10.5 years in the PAL group and 61.9 ± 12.7 years in the non-PAL group; there was no significant difference between the two groups (p = 0.625). Moreover, there were no statistical differences between the two groups with respect to gender, body-mass index, diabetes mellitus, hypertension, cardiovascular disease, preoperative albumin, operative site, or forced expiratory volume-one second (FEV1). Tumor sizes were 1.34 ± 0.78cm and 1.61 ± 1.43cm in the PAL and non-PAL groups, respectively; these were not statistically different (p = 0.442). The mean DLCO value was 97.3% ± 18.3% in the PAL group and 111.7% ± 15.9% in the non-PAL group; this was statistically different (p = 0.014). In addition, there were significant differences between the two groups with respect to EI (4.61% ± 4.66% vs. 1.17% ± 1.76%; p = 0.023) and LEI (5.81% ± 5.78% vs. 0.76% ± 1.17%; p = 0.009), as well as hospital stay (8.9 ± 2.4 days vs. 4.9 ± 1.6 days; p < 0.001) (Table 1).

**Table 1:** Baseline characteristics according to the prolonged air-leak.

Characteristics	PAL (+) (n = 13)	Non-PAL (n = 28)	*p* Value
Age, years	63.9 ± 10.5	61.9 ±12.7	0.625
Sex, male	7 (53.9%)	9 (32.1%)	0.185
BMI, m^2^/kg	23.86 ± 3.54	23.76 ± 2.82	0.913
HTN	7 (53.9%)	12 (42.9%)	>0.999
CVD	1 (7.7%)	1 (3.6%)	0.539
Preoperative albumin, g/dL	4.45 ± 0.29	4.31 ± 0.22	0.111
Tumor size, cm	1.34 ± 0.78	1.61 ± 1.43	0.442
Operation site, Right	3 (23.1)	13 (46.4)	0.154
FEV1, %	113.0 ± 17.5	107.8 ± 17.7	0.377
DLCO, %	97.3 ± 18.3	111.7 ± 15.9	0.014
EI, %	4.61 ± 4.66	1.17 ± 1.76	0.023
LEI, %	5.81 ± 5.78	0.76 ± 1.17	0.009
HD, days	8.9 ± 2.4	4.9 ± 1.6	<0.001

BMI: body mass index; HTN: hypertension; CVD: cardiovascular disease; FEV1: forced expiratory volume in 1 second; DLCO: diffusing capacity for carbon monoxide; EI: emphysema index; LEI: lobe-specific emphysema index; HD: hospital day

The types of segmentectomy were summarized in the Table 2. There was no significant difference between the PAL group and non-PAL group with respect to the type of segmentectomy.

**Table 2:** Comparison of types of segmentectomy.

Characteristics	PAL (+) (n = 13)	Non-PAL (n = 28)	*p* Value
Type of segmentectomy			0.065
Right upper lobe			
Apical segmentectomy	0 (0%)	2 (7.1%)	
Anterior segmentectomy	1 (7.7%)	1 (3.6%)	
Right middle lobe			
Medial segmentectomy	1 (7.7%)	0 (0%)	
Lateral segmentectomy	1 (7.7%)	0 (0%)	
Right lower lobe			
Superior segmentectomy	0 (0%)	7 (25%)	
Basal segmentectomy	0 (0%)	3 (10.7%)	
Left upper lobe			
Trisegmentectomy (upper division)	2 (15.4%)	2 (7.1%)	
Lingular segmentectomy	6 (46.2%)	9 (32.1%)	
Left lower lobe			
Superior segmentectomy	2 (15.4%)	1 (3.6%)	
Basal segmentectomy	0 (0%)	3 (10.7%)	

PAL: Prolonged air leak

**Fig 3:**
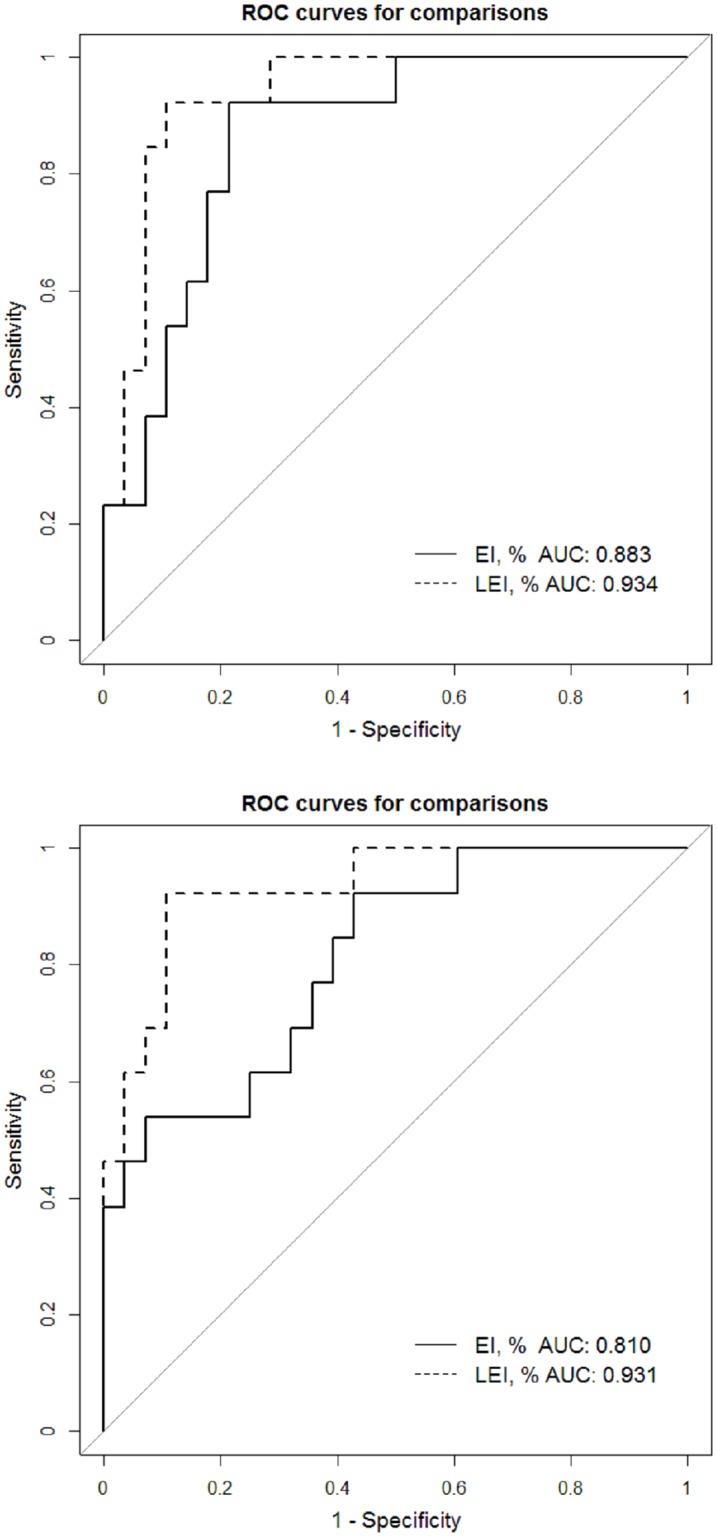
Receiver operating characteristic curves for comparing the emphysema index and lobe-specific emphysema index. Unadjusted analysis (A) and adjusted analysis (B).

**Fig 4:**
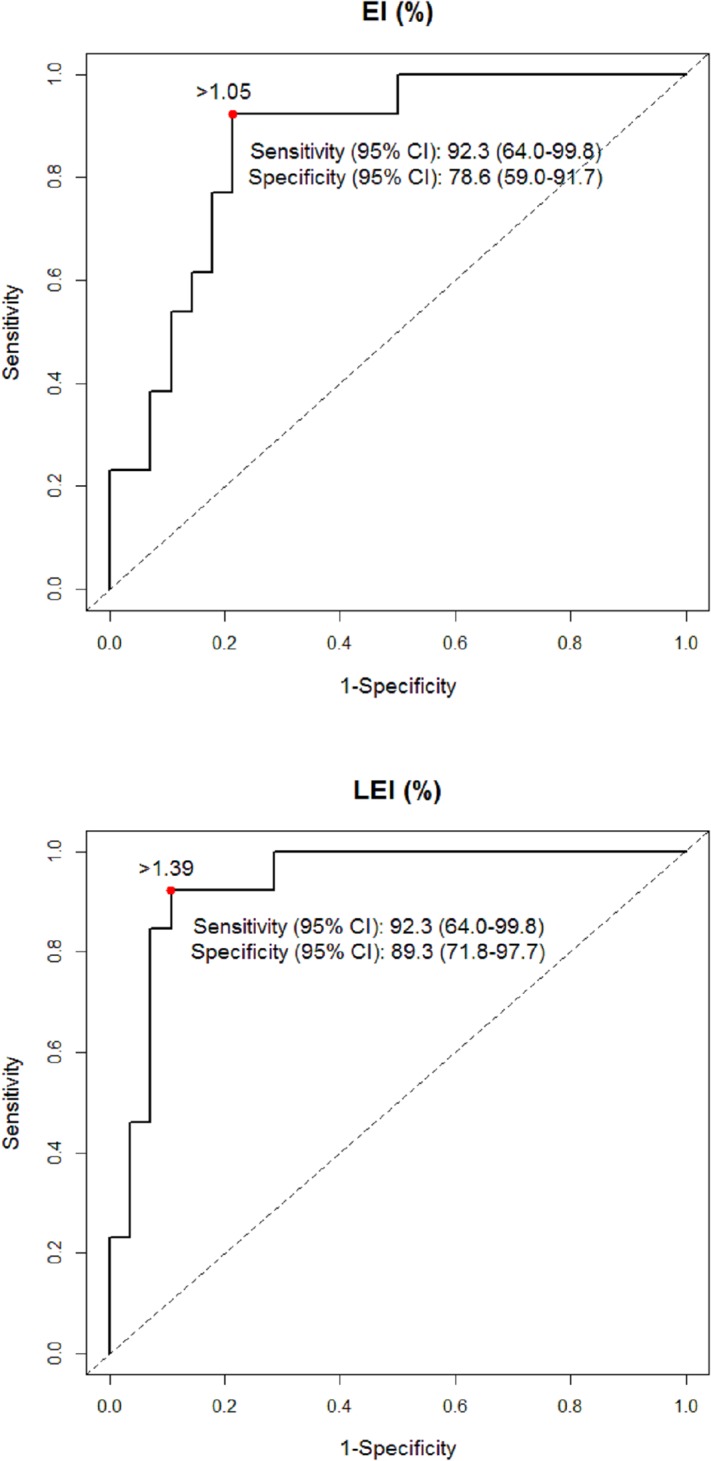
Youden index for determining the optional cutoff value for emphysema index (A) and lobe-specific emphysema index (B).

**Fig 5:**
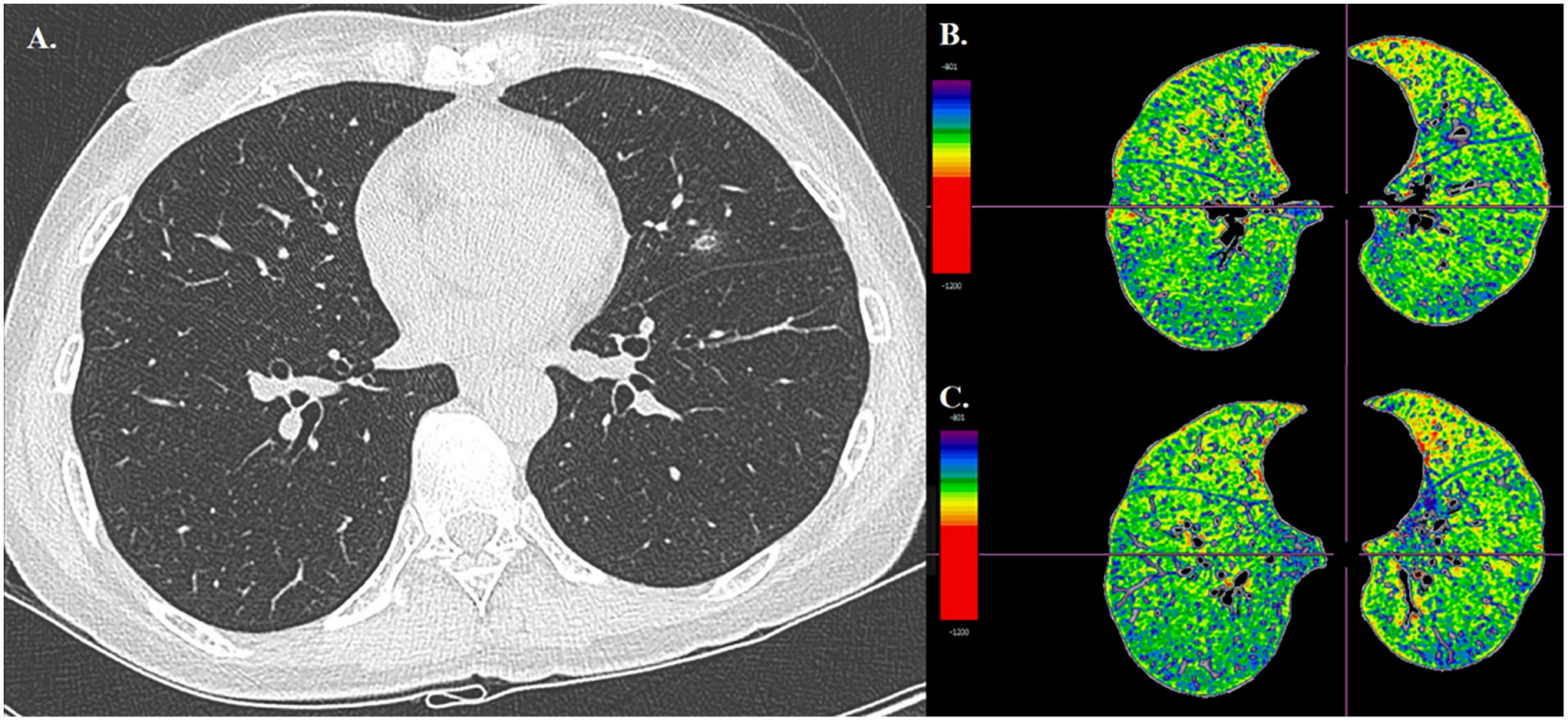
Representative case of prolonged air leak after anatomical segmentectomy. A 54 year-old female patient underwent anatomical segmentectomy for a persistent subsolid nodule approximately 1cm in left lingular segment (A). On pre-operative chest CT images, emphysema index of the entire lung is 11.6% and the lobe-specific emphysema index is 17.8% (B, C). Prolonged air leak occurred after lingular segmentectomy.

According to our correlation analysis, EI was significantly correlated with LEI (r = 0.939; p < 0.001). To minimize multicollinearity in our logistic regression analysis, we used the following two models: model 1 included DLCO and EI, whereas model 2 included DLCO and LEI. Our multivariate analysis showed that both EI and LEI were statistically significant risk factors for PAL [OR, 1.555; 95% CI, 1.049–2.303; p = 0.028; vs. OR, 2.766; 95% CI, 1.372–5.573; p = 0.004] (Table 3). When we compared the two predictors, LEI showed greater predictive power in both unadjusted and adjusted analyses via the DeLong method (Fig 3). The optimal cut-off value for EI was 1.05 (sensitivity 92.3%; specificity 78.6%), while that for LEI was 1.39 (sensitivity 92.3%; specificity 89.3%) (Figs 4 and 5).

**Table 3:** Risk factor for prolonged air-leak in anatomical segmentectomy.

Variable	Univariate Analysis	Multivariable Analysis (Logistic Regression Analysis Model)			
	*p* Value	Model 1		Model 2	
		OR (95% CI)	*p* Value	OR (95% CI)	*p* Value
DLCO	0.024	0.954 (0.906–1.004)	0.069	0.965 (0.902–1.033)	0.308
EI, %	0.019	1.555 (1.049–2.303)	0.028		
LEI, %	0.002			2.766 (1.372–5.573)	0.004

DLCO: diffusing capacity for carbon monoxide; EI: emphysema index; LEI: lobe-specific emphysema index; OR: odds ratio; CI: confidence interval

## Discussion

We hypothesized that LEI may be a specific risk factor for PAL after anatomical segmentectomy. As shown in this study, PAL was strongly influenced by both LEI and EI, suggesting that these factors can indeed predict PAL after anatomical segmentectomy. However, after closer analysis, we determined that LEI had greater predictive power than EI.

Emphysema is defined as anatomical destruction of the lung with abnormal, permanent enlargement of airspaces distal to the terminal bronchiole; this results in an abnormal area with low attenuation on CT images [10]. Müller and colleagues reported good correlation between CT densitometry and pathologic changes in patients with emphysema in 1988; since then, quantification of emphysema on CT images via densitometry has widely been used for diagnosis, characterization, and stratification of emphysema [11]. The HU value of -950 is regarded as the most common threshold for defining an abnormally low attenuation area; voxels with CT attenuation of less than -950 HU are assumed to indicate an emphysematous lung [12–14]. EI is calculated on the basis of whole-lung analysis; it is more commonly used than LEI due to the difficulty of lobe segmentation. However, with improved three-dimensional software and segmentation techniques in daily practice, LEI has become increasingly popular [15]. In this study, we analyzed LEI and EI by simultaneously using a semi-automatic lobe segmentation technique and CT densitometry.

According to Cerfolio et al., important characteristics of PAL after lung resection include factors that influence healing of the lung parenchyma, the presence of adhesion, and the onset of emphysema. They speculated that various techniques–fissureless technique, pleural tenting, buttressed stapling procedure, use of pneumoperitoneum, and focal seal–might influence the development of PAL. Moreover, they showed that lobectomy, when compared with sublobar resection, (similar to wedge resection and segmentectomy), requires greater lung parenchyma resection, which can result in a greater likelihood of PAL; hence, they suggested that PAL may be a larger problem for lobectomy than sublobar resection [3]. However, there has recently been a sharp increase in the detection of early stage lung cancer, due to recent advancements and greater applicability of low-dose chest CT. Given the increased prevalence of early stage lung cancer, there has also been an increase in the number of sublobar resections performed, particularly those involving anatomical segmentectomy [8,16]. Despite this increase, to the best of our knowledge, there have been no studies regarding factors predictive of PAL after anatomical segmentectomy. Hence, our study aimed to analyze the factors that predict the development of PAL after anatomical segmentectomy. In our study, we excluded patients with adhesion and those using steroids; we included only those who underwent VATS to eliminate all factors that may affect the healing of lung parenchyma. Petrella et al. found that the best predictor of PAL after lobectomy is CT quantification of emphysema; consistent with this, we evaluated whether EI and LEI can predict PAL after segmentectomy [7].

Identifying the anatomical intersegmental plane is a difficult task when performing segmentectomy via minimally invasive surgery, such as VATS [17,18]. If the tumor is larger than 2 cm in size, or if it is located close to the intersegmental plane, thoracic surgeons tend to resect more distally from the intersegmental plane during division; this ensures a sufficient and safe resection margin, especially when optimizing the preservation of lung function [19,20]. However, this is not a simple surgical technique. Therefore, to perform these procedures, resection will inevitably require additional manipulation of the specific lobe, which ultimately increases the likelihood of PAL. Hence, as hypothesized, LEI was more robust than EI in predicting the development of PAL.

PAL is one of the most common complications after lung resection [1–4].; notably, it also increases the likelihood of developing other serial complications. Thus, it is often associated with prolonged hospital stay and increased hospital costs, as well as significant physical and psychological burden [1–4]. Therefore, if PAL can be predicted preoperatively, surgeons may implement various additional tools during the operation [4,21–23]. In our study, we did not use any fibrin glue or other additional surgical material to cover the injured lung parenchyma or resected segmental plane. However, our findings indicate that additional, active procedures are necessary during the operation for patients with high LEI who are undergoing segmentectomy.

There were several limitations to this study. First, this study was a single-center, retrospective study with a small number of patients. Second, this study included three surgeons, rather than one; all three performed anatomical segmentectomy for patients in this study, which likely resulted in procedural differences that may have affected the results. Third, PAL was defined as patients with air leak for more than 5 consecutive days, as well as those with a history of chemical pleurodesis. Hence, the incidence of PAL in this study may be higher than the average occurrence after segmentectomy.

In conclusion, we found that EI and LEI are both significant factors that can predict the development of PAL after anatomical segmentectomy. However, LEI appears to be more robust as a predictor of PAL. We believe that this is a meaningful finding, especially given the relative ease with which the values of EI and LEI could be obtained via a three-dimensional software package. A future study with a larger cohort is necessary to better understand these findings.

## Supporting information

S1 File Attached file includes data of the baseline characteristics, emphysema indices and lobe-specific emphysema indices. (XLSX)
